# Spermidine-Regulated Biosynthesis of Heat-Stable Antifungal Factor (HSAF) in *Lysobacter enzymogenes* OH11

**DOI:** 10.3389/fmicb.2018.02984

**Published:** 2018-12-04

**Authors:** Yuan Chen, Lingjun Yu, Fengquan Liu, Liangcheng Du

**Affiliations:** ^1^Institute of Plant Protection, Jiangsu Academy of Agricultural Sciences, Nanjing, China; ^2^Department of Chemistry, College of Arts and Sciences, University of Nebraska-Lincoln, Lincoln, NE, United States

**Keywords:** *Lysobacter enzymogenes*, HSAF, spermidine, *S*-adenosylmethionine decarboxylase, arginine decarboxylase, arginase

## Abstract

Heat-Stable Antifungal Factor (HSAF) and its analogs are antifungal natural products produced by the biocontrol agent *Lysobacter enzymogenes*. The production of HSAF is greatly influenced by environmental stimuli and nutrients, but the underlying molecular mechanism is mostly unclear. Here, we found that HSAF production in *L. enzymogenes* OH11 is strictly controlled by spermidine, which is the most prevalent triamine in bacteria. When added into OH11 cultures, spermidine regulated the production of HSAF and analogs in a concentration-dependent manner. To verify the role of spermidine, we deleted *Le*SDC and *Le*ADC genes, encoding *S*-adenosylmethionine decarboxylase and arginine decarboxylase, respectively, that are the key enzymes for spermidine biosynthesis. Both deletion mutants produced barely detectable spermidine and HSAF including its analogs, whereas the antifungals production was restored by exogenous spermidine. The results showed that the OH11 cells must maintain a proper spermidine homeostasis for the antifungals production. Indeed, the expression level of the key HSAF biosynthetic genes was significantly impaired in *Le*SDC and *Le*ADC mutants, and exogenous spermidine restored the gene expression level in the mutants. Ornithine is a key substrate for HSAF biosynthesis, and OH11 genome contains *arg1* and *arg2* genes, encoding arginases that convert arginine to ornithine. While the expression of *arg1* and *arg2* was affected slightly upon mutation of *Le*SDC and *Le*ADC, exogenous spermidine significantly increased the arginase gene expression in *Le*SDC and *Le*ADC mutants. Together, the data revealed a previously unrecognized mechanism, in which spermidine controls antibiotic production through controlling both the biosynthetic genes and the substrate-production genes.

## Introduction

For decades, the Gram-positive actinomycetes have been the main source for bioactive natural products (Genilloud, 2017). Many Gram-negative bacteria, such as the ubiquitous environmental microorganisms *Lysobacter*, remain largely underexplored (Xie et al., 2012). We previously isolated Heat-Stable Antifungal Factor (HSAF) and its analogs, a group of polycyclic tetramate macrolactams (PoTeMs), from *L. enzymogenes* strains (Yu et al., 2007). HSAF shares the same chemical structure with dihydromaltophilin, and its absolute configuration was first established in 2015 (Graupner et al., 1997; Nakayama et al., 1999; Xu et al., 2015; Puopolo et al., 2018). So far, about a dozen of analogs/precursors of HSAF have been reported (Li et al., 2018). HSAF exhibits inhibitory activity against a wide range of fungal species, and its chemical structure and mode of action are distinct from existing antifungal drugs or fungicides (Li et al., 2006; Ding et al., 2016a,b). Attempts to apply these antifungal compounds, however, for pharmaceutical purposes and biological control of plant diseases have been challenging because production of HSAF and its analogs is greatly influenced by environmental stimuli and nutrients. For example, HSAF and analogs are produced only in the nutrient-limited media, such as 1/10 tryptic soy broth (TSB) medium (Doyle et al., 1968; Yu et al., 2007). As the underlying molecular mechanism is not well understood, the knowledge of how biosynthesis of the antifungals is regulated in *L. enzymogenes* could facilitate their applications.

Previous studies have identified multiple factors effecting HSAF biosynthesis in *L. enzymogenes*. For example, the global transcriptional regulator Clp is essential for HSAF production in *L. enzymogenes* (Qian et al., 2013; Wang et al., 2014). The two-component systems (TCSs), *Le*-RpfC/*Le*-RpfG and *Le*-QseC/*Le*-QseB, are involved in signal transduction of the small molecule fatty acid *Le*DSF3 and indole, respectively, both of which affect HSAF biosynthesis (Han et al., 2015, 2017). In addition, evidences showed that another two response regulators, PilG and PilR are involved in the regulation of HSAF biosynthesis (Zhou et al., 2015; Chen et al., 2017b). LesR (a solo LuxR regulator) and LetR (a TetR-family protein) are negative regulators of HSAF biosynthesis in *L. enzymogenes* (Qian et al., 2014; Wang et al., 2017; Xu et al., 2017). In addition to *Le*DSF3 and indole, a third metabolite, 4-hydroxybenzoic acid (4-HBA) was found to serve as a diffusible factor regulating HSAF biosynthesis via LysR_Le_, a LysR-type transcription factor (Su et al., 2017). A recent study identified another transcription factor LarR, a member of the MarR-family proteins and suggested a cross-talk between LysR_Le_ and LarR during 4-HBA-regulated HSAF production (Su et al., 2018). The previous studies have drawn a hierarchical network of HSAF regulation, in which multiple protein regulators are involved in controlling HSAF production in *L. enzymogenes*. Considering the diverse habitat of *Lysobacter* species, the presence of a complex regulatory network is essential for the survival of the bacteria in dynamic environments. To date, *Le*DSF3, indole and 4-HBA are the only characterized small molecule signals in *L. enzymogenes*. The involvement of multiple regulatory proteins in HSAF biosynthesis suggests that there still exist yet-to-be-recognized important small molecule signals in *L. enzymogenes*. These small molecules could be environmental stimuli/nutrients or intracellular metabolites.

**FIGURE 1:**
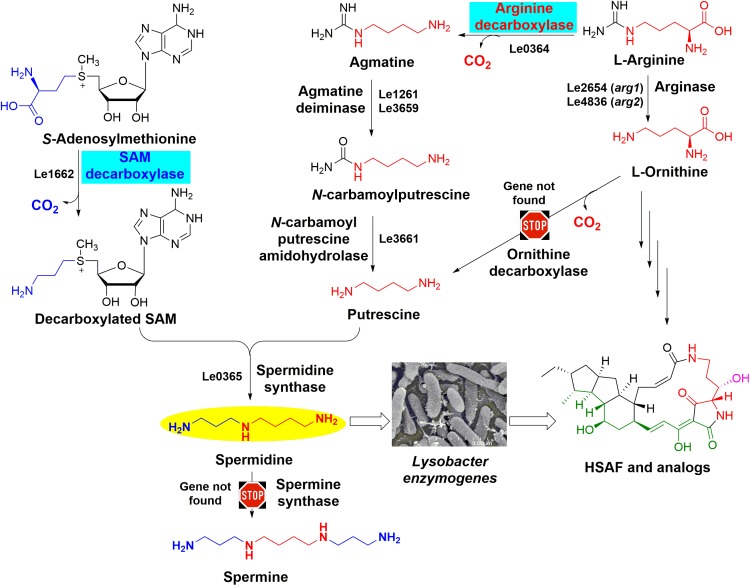
The predicted spermidine biosynthesis pathway in *Lysobacter enzymogenes* OH11. The two key steps catalyzed by arginine decarboxylase and *S*-adenosylmethionine decarboxylase are highlighted. The genes that were predicted to encode the enzymes for spermidine biosynthesis (such as *Le0364* for ADC and *Le1662* for SDC) are included for each of the steps, except the genes for ornithine decarboxylase and spermine synthase due to the lack of the genes in OH11 genome. Thus, putrescine and spermidine are only derived from arginine in OH11, and spermine is not present in OH11. The GenBank accession numbers of the genes encoding proteins involved in spermidine biosynthesis are MH718925 for *Le0364*, MH718926 for *Le1261*, MH718927 for *Le3659*, MH718930 for *Le3661*, MH718924 for *Le1662*, and MH718931 for *Le0365*. There are two arginases, encoded by Le2654 (*arg1*, GenBank accession number MH718928) and Le4836 (*arg2*, GenBank accession number MH718929), respectively, in the genome of *L. enzymogenes* to covert arginine to ornithine which is the key substrate for HSAF biosynthesis.

In this work, we found that spermidine plays an essential role in HSAF production in *L. enzymogenes* (Figure 1). Spermidine is a naturally occurring polyamine with a variety of biological functions (Xie et al., 2014; Chen et al., 2017a; Madeo et al., 2018; Park et al., 2018). Together with the diamine putrescine and the tetraamine spermine, these polycationic small molecules bind and stabilize polyanionic macromolecules such as DNA and RNA, modulate activity of enzymes, help maintain general homeostasis of cells, and are essential for cell growth and proliferation (Miller-Fleming et al., 2015; Michael, 2016b; Madeo et al., 2018). Our results showed that the mechanism for spermidine to control HSAF production is distinct from the previously reported, protein regulator-mediated mechanisms. The data supported that the spermidine homeostasis in *L. enzymogenes* cells may control the biosynthesis of HSAF and analogs through affecting a key substrate of the antifungals, in addition to controlling the key biosynthetic gene expression level.

## Materials and Methods

### Bacterial Strains and Plasmids

The list of bacterial strains and plasmids used in this study is presented in Table 1. *Lysobacter enzymogenes* strain OH11 was used as the wild type for production of spermidine, HSAF and its analogs (Qian et al., 2009). OH11 and the derived strains were cultured at 30°C in media depending on the purpose of the cultures. When required, antibiotics were added at the final concentration of 50 μg/ml kanamycin (Km) or 150 μg/ml gentamicin (Gm). *Escherichia coli* strain DH5α was used as the host for plasmid maintenance and routinely grew at 37°C in Luria Broth (LB; Bertani, 2004) medium supplemented with appropriate antibiotics according to the antibiotic resistance of plasmids.

**Table 1:** Bacterial strains and plasmids used in this study.

	Relevant genotype ^a^	Source
**Strains**		
**Lysobacter enzymogenes**		
OH11	Wild type, Km^R^	Qian et al., 2009; CGMCC No. 1978
**In-frame deletion mutants**		
ΔADC	In-frame deletion of *Le0364*, Km^R^	This study
ΔSDC	In-frame deletion of *Le1662*, Km^R^	This study
ΔArg1	In-frame deletion of *Le2654*, Km^R^	This study
ΔArg2	In-frame deletion of *Le4836*, Km^R^	This study
ΔArg1–ΔArg2	In-frame deletion of *Le2654* and *Le4836*, Km^R^	This study
**Escherichia coli**		
DH5α	Host strain for molecular cloning	Chen et al., 2018
**Plasmids**		
pEX18GM	Suicide vector with *sacB* and Gm^R^	Hoang et al., 1998
ADC-pEX18	pEX18GM with two flanking fragments of *Le0364*, Gm^R^	This study
SDC-pEX18	pEX18GM with two flanking fragments of *Le1662*, Gm^R^	This study
pJQ200SK	Suicide vector with *sacB* and Gm^R^	Quandt and Hynes, 1993
Arg1-pJQ200SK	pJQ200SK with two flanking fragments of *Le2654*, Gm^R^	This study
Arg2-pJQ200SK	pJQ200SK with two flanking fragments of *Le4836*, Gm^R^	This study


*^a^ Km^R^ and Gm^R^, kanamycin- and gentamicin-resistant, respectively.*

### Construction of In-Frame Gene Deletion Mutants of *L. enzymogenes*

The whole genome of *L. enzymogenes* strain OH11 was sequenced, assembled and annotated by BGI company (Shenzhen, China). The sequences of enzymes related to spermidine biosynthesis reported previously, such as ornithine decarboxylase (ODC, EC 4.1.1.17), arginine decarboxylase (ADC, EC 4.1.1.19), agmatine deiminase (EC 3.5.3.12), *N*-carbamoyl putrescine amidohydrolase (NCPAH, EC 3.5.1.53), *S*-adenosylmethionine decarboxylase (SDC, EC 4.1.50), spermidine synthase (SpdSyn, EC 2.5.1.16), spermine synthase (SpmSyn, EC 2.5.1.22) and arginase (EC 3.5.3.1), were downloaded from NCBI protein database using the EC number (Michael, 2016a). Then BLASTP analysis was performed against the genome of *L. enzymogenes*. The downloaded sequences were used as subject proteins to identify spermidine biosynthesis related proteins in *L. enzymogenes*. The deletion mutants in *L. enzymogenes* were constructed as previously described (Qian et al., 2012). The plasmids pEX18Gm or pJQ200SK were used to obtain the deletion mutants. First, the flanking regions of the target gene were amplified by PCR using the primers listed in Table 2 and assembled into the corresponding restriction sites of vector to construct the recombinant plasmid. Phusion High-Fidelity DNA polymerase (Thermo Scientific) was used for PCR experiments in this study according to the following 3-step program: 98°C for 30 s at the initial denaturation step; 30 cycles (98°C for 10 s, 60°C for 15 s, 72°C for 1 min) of denaturation annealing step; and 72°C for 5 min in the final extension step. The construct was sequenced by Eurofins MWG Operon LLC to confirm the fidelity of the inserted fragments. Then the correct recombinant plasmid constructs were transformed into the wild-type strain OH11 by electroporation (Eppendorf Eporator, Eppendorf North America, Inc.). The single-crossover recombinants were selected on LB plates supplemented with Km and Gm. The Gm-resistant colonies were cultured for 6 h in liquid LB medium without antibiotics for double-crossover recombinants enrichment. Subsequently, the recombinants were counter-selected on LB plates containing 10% (w/v) sucrose to screen for colonies lost the original vector. Finally, the sucrose-resistant, Km-resistant, but Gm-sensitive colonies were picked and verified by PCR using appropriate primers listed in Table 2.

**Table 2:** Primers used in this study.

Primer	Sequence	Purpose
**Primers used for gene in-frame deletion**		
ADC-uF	GGGGTACCCTGGCAGACGCTTTACTCGC (*Kpn*I)	To amplify 1,131-bp upstream homolog arm of *Le0364*
ADC-uR	CCCAAGCTTGGAAGGCGCATTCTACAGG (*Hin*dIII)	
ADC-dF	CCCAAGCTTCCGCCTACCACGCCAAGGTC (*Hin*dIII)	To amplify 888-bp downstream homolog arm of *Le0364*
ADC-dR	GCTCTAGAGCGACACGCACAGCACCA (*Xba*I)	
SDC-uF	GGGGTACCACGACATAATAGAGGGTGCTGG (*Kpn*I)	To amplify 450-bp upstream homolog arm of *Le1662*
SDC-uR	CCCAAGCTTAGGGCCTTGGTGAGGTTGT (*Hin*dIII)	
SDC-dF	CCCAAGCTTTCTACCACGGGCGCAATCTG (*Hin*dIII)	To amplify 431-bp downstream homolog arm of *Le1662*
SDC-dR	GCTCTAGAGTGCTCGAACACTGCGGCTA (*Xba*I)	
SDC-vF	TCCTCTTCCTCGGTAATGATGC	To confirm mutant construction of ΔSDC
SDC-vR	ATCGGGTCGGTCTTGGCGGTAC	
Arg1-uF	CGGGGCCCCATTGGAACGACAGCCTCTT (*Apa*I)	To amplify 981-bp upstream homolog arm of *Le2654*
Arg1-uR	CCGCTCGAGCGGCAAGACAGGGGAAGA (*Xho*I)	
Arg1-dF	CCGCTCGAGGACTTGGTCGAGAGCCTG (*Xho*I)	To amplify 998-bp downstream homolog arm of *Le2654*
Arg1-dR	GGACTAGTGGCATTCCCCTCTTGTGA (*Spe*I)	
Arg2-uF	GGACTAGTCCCTCATCGTCCTGTTGG (*Spe*I)	To amplify 1,504-bp upstream homolog arm of *Le4836*
Arg2-uR	CCGCTCGAGAACGAGGGGATAAGTGCG (*Xho*I)	
Arg2-dF	CCGCTCGAGAGCCTGTTCGGCAAGTCG (*Xho*I)	To amplify 1,416-bp downstream homolog arm of *Le4836*
Arg2-dR	CGGGGCCCGTTCGTATCGGCGTTGGC (*Apa*I)	
**Primers used for qRT-PCR**		
*pks*-RT-F	ACTATTTGTTGGGCGACGAC	
*pks*-RT-R	GTAACCGAACAGGGTGCAA	
*arg1*-RT-F	GTCATCGCCTGGAACCG	
*arg1*-RT-R	TCGTTGGTGTTGAAGTCGG	
*arg2*-RT-F	GAAGGCGTGGACGAGAACA	
*arg2*-RT-R	GAAGGCGTGGACGAGAACA	
16S-RT-F	ACGGTCGCAAGACTGAAACT	
16S-RT-R	AAGGCACCAATCCATCTCTG	


### Extraction and Detection of HSAF and Analogs

The strains were cultured in 25 ml of 1/10 TSB medium for 48 h. Before extraction, 75 μl of trifluoroacetic acid (TFA) were added into the cultures to adjust the pH. HSAF and analogs were extracted with 30 ml ethyl acetate. The upper organic phase was collected and evaporated, and the residues were dissolved in 300 μl methanol containing 0.05% TFA (v/v). A 20 μl aliquot was analyzed by HPLC (Agilent, 1220 Infinity LC). HSAF and analogs were eluted by mobile phase A (water, 0.05% TFA) and mobile phase B (acetonitrile, 0.05% TFA). The elution started from 5% B and increased to 25% in the first 5 min, linearly increased to 80% in the following 20 min, increased to 100% in 3 min, and then returned to 5% in the rest 2 min. The production of HSAF and analogs was normalized using the ratio of corresponding peak area and optical density at 600 nm (OD_600_). Three biological replicates were conducted for each sample, and each was assayed in three technical replicates.

### Spermidine Extraction, Derivatization, and Detection by HPLC

The extraction, derivatization and detection methods of intracellular spermidine were carried out according to previously described protocols with modifications (Li et al., 2016; Sakanaka et al., 2016). Briefly, the strains were cultured in 25 ml 1/10 TSB medium for 48 h. A 2 ml fraction of the cultures was centrifuged at 15,000 rpm for 3 min at 4°C to collect the cells. The pelleted cells were washed three times with phosphate-buffered saline (PBS) buffer and centrifuged at 15,000 rpm for 5 min at 4°C. The washed cells were resuspended in 250 μl 5% (v/v) trichloroacetic acid (TCA) and incubated in boiling water for 15 min. After centrifuging at 15,000 rpm for 5 min at 4°C, a 200 μl fraction of supernatants was transferred into a new 2 ml Eppendorf tube and kept for further derivatization. For polyamine derivatization, 200 μl of NaOH (10 M) solution and 10 μl of benzoyl chloride (open under a stream of N_2_) were added into the above extracts. The solutions were mixed by vigorously shaking for 2 min and stood for 20 min at room temperature. A 200 μl of saturated NaCl was added into the mixture and vortexed for 2 min. Subsequently, the resulting derivatives were extracted twice with 1.5 ml diethyl ether each time. The upper organic phase was combined and kept in a fume hood until fully evaporated under air. The residues in the tube were kept at -20°C for further analysis. The benzoylated polyamine extracts were analyzed with a C18 column (Agilent Eclipse XDB-C18 column, 250 × 4.6 mm, 3.5 μm) using Agilent HPLC (1220 Infinity LC), equipped with a UV detector set at 254 nm. Benzoylated polyamines were eluted by mobile phase A (water containing 0.05% TFA) and mobile phase B (acetonitrile containing 0.05% TFA) using the following gradient elution program: mobile phase B was increased from 5 to 20% in the first 5 min, continued to increase till 50% in the following 5 min and maintained for 12 min, then increased to 100% during 22–26 min, and returned to 5% in the final 5 min. Before injection, the benzoylated samples were dissolved in 250 μl of methanol and centrifuged at 13,000 rpm for 10 min. An aliquot of 20 μl was injected for HPLC analysis.

To quantify the intracellular spermidine concentration, a calibration curve with spermidine concentrations ranging from 10 to 500 μM was established by HPLC analysis. Meantime, the total intracellular protein concentration in the respective cultures was measured according to the manual of Pierce^TM^ BCA protein assay kit (Thermo Fisher), with bovine serum albumin as the standard. Finally, the normalized concentration of intracellular spermidine was determined as nmol/μg cellular protein. The data were derived from three independent experiments with triplicate samples.

### RNA Extraction and qRT-PCR

The bacterial strains were cultured in 1/10 TSB or 1/10 TSB supplemented with various concentrations of spermidine. The cultures were allowed to grow for 12 h and the cells were collected. The primers used for qRT-PCR are listed in Table 2. The method for RNA extraction and qRT-PCR was essentially identical to that described previously (Yu et al., 2018). The data were derived from three independent experiments with triplicate samples.

### Statistical Analysis

Statistical analysis was performed by IBM SPSS 14.0 software (SPSS Inc., Chicago, IL, United States) using the unpaired Dunnett’s *t*-test (*P* < 0.05) to calculate significant differences.

## Results and Discussion

### Exogenous Supplement of Spermidine Inhibited Production of HSAF and Analogs in *L. enzymogenes*

**FIGURE 2:**
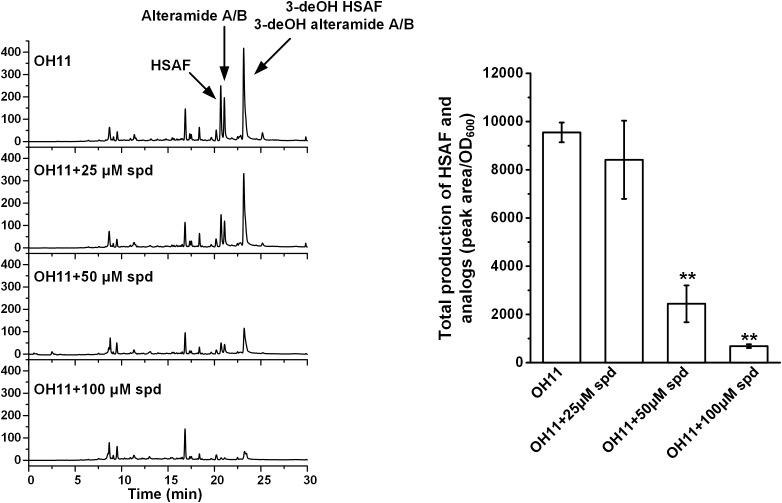
HPLC analysis of production of HSAF and analogs in wild-type OH11, without or with exogenously supplemented spermidine (spd, 25–100 μM). The quantitative analysis of HSAF and analogs is shown in the right panel (^∗∗^*P* < 0.01). The data were derived from three independent experiments with triplicate samples.

Polyamines are essential polycations found throughout all kingdoms of life, and spermidine is the main triamine in bacteria (Michael, 2016b). Since these small molecules function in many cellular processes, including gene expression and regulation, protein translation, autophagy, and stress response (Gevrekci, 2017), we figured that spermidine could be important for the production of HSAF and analogs in *L. enzymogenes*. The wild-type strain OH11 produces a series of metabolites belonging to the PoTeM family, including HSAF, alteramide A/B, 3-dehydroxy HSAF, and 3-dehydroxy alteramide A/B (Li et al., 2018). When the OH11 culture was exogenously supplemented with spermidine (25–100 μM), the yield of the PoTeM compounds decreased gradually, with only approximately 5% of the wild type level when exogenously added spermidine concentration reached 100 μM (Figure 2).

As shown in Figure 1, the cellular spermidine level depends on the overall balance of uptake, export, biosynthesis, and catabolism of polyamines (Kusano et al., 2008). To estimate the spermidine level in OH11 cells, we extracted polyamines from OH11 cells and analyzed by HPLC. In the wild-type strain OH11 grown in 1/10 TSB which is the typical HSAF production medium (Yu et al., 2007), spermidine was clearly the dominant polyamine, with a level of about 0.08 nmol/μg cellular proteins (Supplementary Figure S1). The other polyamines, such as spermine and putrescine, were not detectable. When a low concentration (25 μM) of exogenous spermidine was added into the OH11 culture, the detectable level of cellular spermidine actually dropped slightly; when a higher concentration (50–100 μM) of exogenous spermidine was added, the cellular spermidine level increased slightly (over 0.1 nmol/μg cellular proteins). However, the degree of increase was not proportional to the concentration of exogenous spermidine added, indicting the buffering ability of OH11 cells to balance the cellular concentration of polyamines. Interestingly, when the wild-type strain OH11 was allowed to grow in full TSB, in which very little HSAF was produced (Yu et al., 2007), the cellular concentration of spermidine, about 0.01 nmol/μg cellular proteins, was clearly lower than that in 1/10 TSB. The results suggested that even a slight change of the cellular polyamine homeostasis can result in a significant change in the production of HSAF and analogs in *L. enzymogenes* OH11.

### Deletion of Spermidine Biosynthetic Genes Eliminated the Production of HSAF and Analogs in *L. enzymogenes*

As shown in Figure 1, spermidine is biosynthesized from putrescine and *S*-adenosylmethionine (SAM) (Murray-Stewart et al., 2016). In many organisms, ornithine is converted directly into putrescine upon a decarboxylation, which is catalyzed by ornithine decarboxylase. Alternatively, putrescine can be biosynthesized from arginine indirectly (Michael, 2016a). Arginine is first converted to agmatine by ADC, agmatine to *N*-carbamoylputrescine by agmatine deiminase, and putrescine is formed from *N*-carbamoylputrescine by an amidohydrolase (Kusano et al., 2008; Shah and Swiatlo, 2008). To form spermidine, SAM undergoes a decarboxylation, which is catalyzed by SAM decarboxylase. The decarboxylated SAM serves as a carbon electrophile to donate the aminopropyl group to the nucleophilic amine of putrescine, resulting in the triamine spermidine. This step is catalyzed by spermidine synthase (Miller-Fleming et al., 2015). In some organisms, spermidine can be further converted to spermine by adding another aminopropyl group from decarboxylated SAM, which is catalyzed by spermine synthase (Miller-Fleming et al., 2015). To understand how the biosynthesis of spermidine is related to the biosynthesis of HSAF and analogs, we searched the genes relevant to polyamine biosynthesis in the genome of OH11 (GenBank accession number: RCTY00000000). Interestingly, OH11 genome does not appear to contain genes encoding the ODC or spermine synthase. This is consistent with the above result that spermine is not detectable in OH11. The genomic information also implies that putrescine and spermidine in OH11 are derived from arginine, not from ornithine (Figure 1).

**FIGURE 3:**
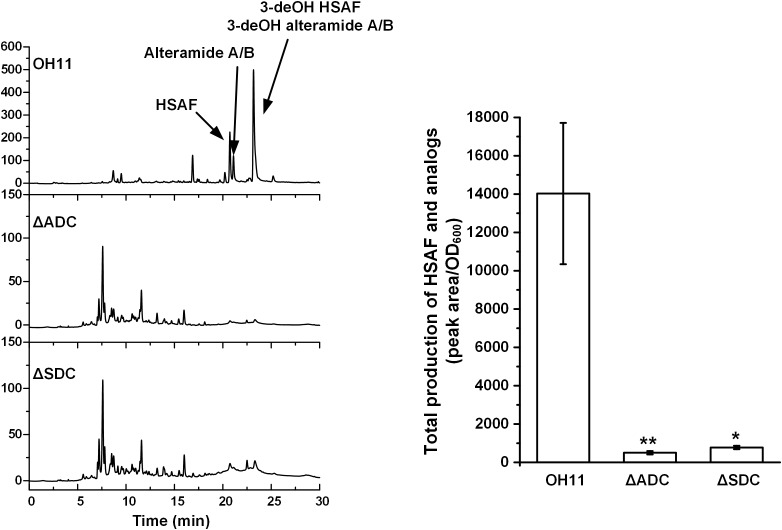
HPLC analysis of HASF and analogs in wild-type OH11, deletion mutant of gene encoding arginine decarboxylase (ΔADC), and deletion mutant of gene encoding SAM decarboxylase (ΔSDC). The quantitative analysis of HSAF and analogs is shown in the right panel (^∗^*P* < 0.05, ^∗∗^*P* < 0.01). The data were derived from three independent experiments with triplicate samples.

**FIGURE 4:**
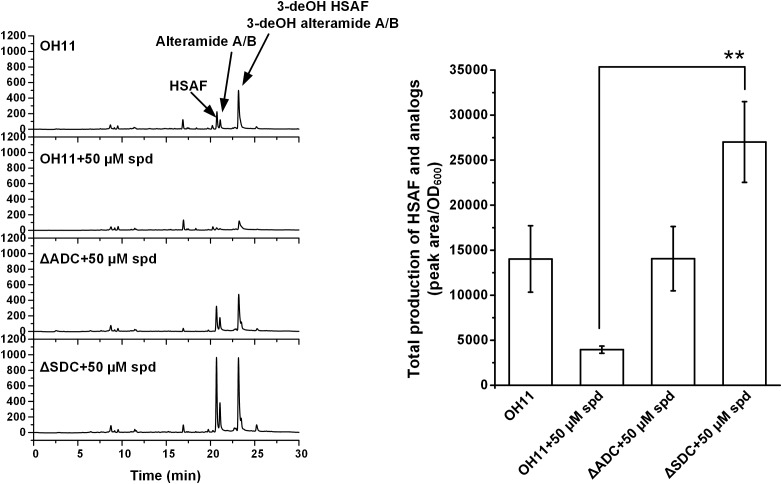
HPLC analysis of HSAF and analogs extracted from wild-type OH11, as well as ΔADC and ΔSDC mutants treated with exogenous spermidine (spd, 50 μM). Quantitative analysis is shown in the right panel. The data were derived from three independent experiments with triplicate samples. ^∗∗^*P* < 0.01.

Next, we in-frame deleted genes encoding ADC and SDC, respectively, for spermidine biosynthesis in OH11. The resulting mutants, ΔADC and ΔSDC, produced a barely detectable amount of spermidine (Supplementary Figure S2). Coincidently, mutants ΔADC and ΔSDC produced a barely detectable amount of HSAF and analogs (Figure 3). When ΔADC and ΔSDC mutants were exogenously supplemented with spermidine (50 μM), the cellular level of spermidine became detectable and comparable to that of the wild type (Supplementary Figure S2). Correspondingly, the yield of HSAF and analogs in ΔADC was comparable to that of OH11, and the yield in ΔSDC surpassed that of OH11, when the mutants were exogenously supplemented with 50 μM of spermidine (Figure 4). The data demonstrated that maintaining a proper level of spermidine is essential for OH11 to produce HSAF and analogs.

### Mutation of Spermidine Biosynthetic Genes Lowered the Expression Level of HSAF Biosynthetic Gene

**FIGURE 5:**
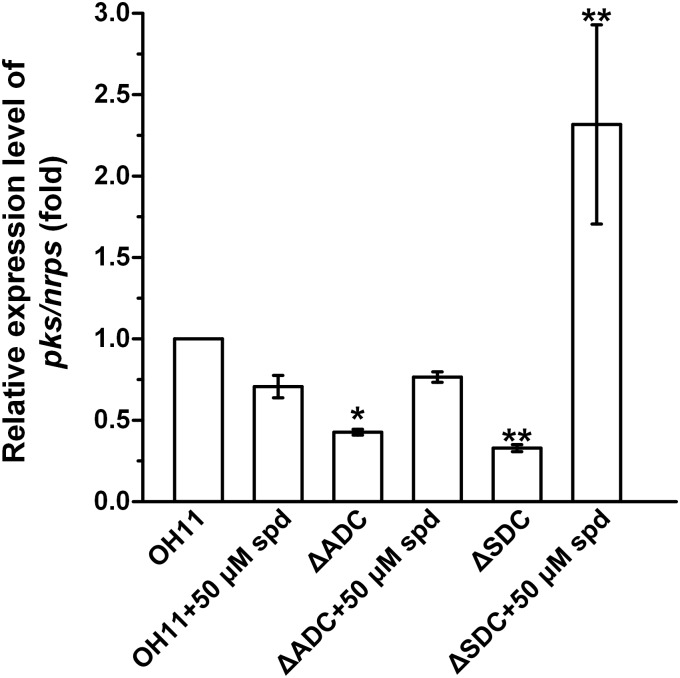
Relative expression level of the key biosynthetic gene *hsaf pks*/*nrps* for HSAF and analogs in wild-type OH11, ΔADC and ΔSDC mutants with or without exogenous spermidine. The transcription level of *pks/nrps* in the wild-type strain OH11 was set as 1. ^∗^*P* < 0.05, ^∗∗^*P* < 0.01.

After having established the connection between spermidine homeostasis and production of HSAF and analogs in OH11, we examined if spermidine executes its effect through affecting the expression level of HSAF biosynthetic genes. The results showed that the expression level of the key biosynthetic gene (*hsaf pks*/*nrps*) for HSAF and analogs in ΔADC and ΔSDC mutants was lower than that in OH11 (Figure 5). Upon addition of exogenous spermidine (50 μM), the expression of *hsaf pks*/*nrps* in ΔADC returned to the wild type level, whereas the expression in ΔSDC was significantly higher than that in the wild type. The data supported that spermidine can control the biosynthesis of HSAF and analogs at the gene transcription level. However, the mutation of the spermidine genes in ΔADC and ΔSDC did not lead to a shutdown of the HSAF biosynthetic gene, although the production of HSAF and analogs was nearly completely shut down in ΔADC and ΔSDC. The results implied that there may be other mechanisms, in addition to the expression control of HSAF biosynthetic gene, accounted for the observed block of HSAF production in the mutants.

### Mutation of Spermidine Biosynthetic Genes Affected Arginase Gene Expression

In many organisms, ornithine decarboxylase is the limiting factor for the biosynthesis of putrescine, spermidine and spermine (Miller-Fleming et al., 2015). Interestingly, no ODC gene was identified in the genome of OH11 (Figure 1) and therefore spermidine in OH11 was possibly derived only from arginine, but not ornithine. However, arginine can also be converted to ornithine through the urea cycle and the enzyme arginase is responsible for this conversion (Morris, 2002). Because ornithine is a key substrate of all HSAF derivatives (Yu et al., 2007; Lou et al., 2011), it would be interesting to examine if there is a connection between the arginine-derived spermidine pathway and the arginine-derived ornithine pathway.

**FIGURE 6:**
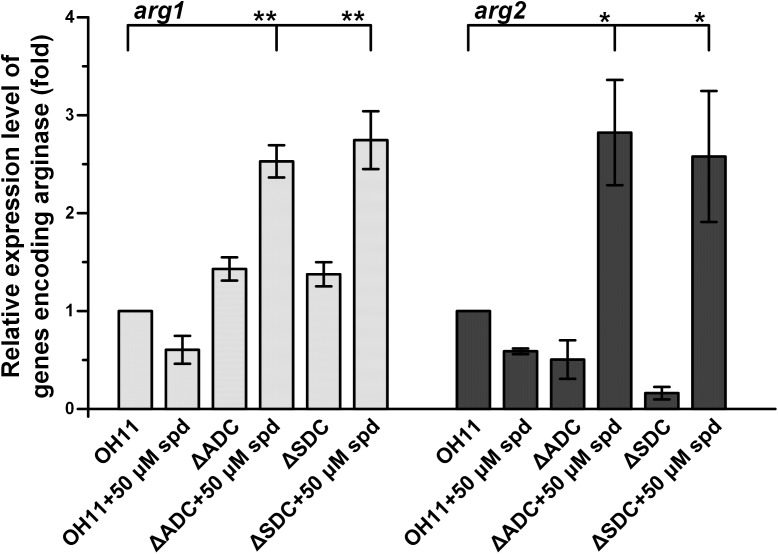
Relative expression level of genes *arg1* and *arg2*, whose encoding products are arginases, in wild-type OH11, ΔADC and ΔSDC mutants treated with or without exogenous spermidine. The transcription level of *arg1* and *arg2* in the untreated wild-type OH11 was set as 1. ^∗^*P* < 0.05, ^∗∗^*P* < 0.01.

In OH11 genome, we identified two homologous genes predicted to encode arginases, *arg1* (*Le2654*) and *arg2* (*Le4836*). We measured the expression level of these two genes in the wild type and spermidine biosynthetic mutants. First, we checked the expression level of *arg1* and *arg2* in the wild type and found that the addition of spermidine (50 μM) to OH11 decreased the transcription of both *arg1* and *arg2*. The results were in parallel with the observed decrease of production of HSAF and analogs in OH11 upon addition of spermidine (Figure 2). In the spermidine mutants, the expression level of *arg2* was slightly decreased by mutation of ADC gene or SDC gene, while the expression of *arg1* was slightly increased by the mutations (Figure 6). The results showed that the arginase genes were affected differently upon the block of the spermidine biosynthesis. However, the addition of exogenous spermidine (50 μM) nearly doubled the *arg2* expression in both mutants, when compared to the wild type. For *arg1* gene, although it was not significantly affected by either ADC mutation or SDC mutation, the addition of exogenous spermidine (50 μM) significantly stimulated *arg1* expression in both mutants (Figure 6). In the wild type, exogenous spermidine exhibits an inhibitory effect on HSAF production, which is consistent with the decrease of expression of the HSAF biosynthetic gene (*hsaf pks*/*nrps*) and the arginase genes (*arg1* and *arg2*); in the spermidine mutants (ΔADC and ΔSDC), exogenous spermidine restores/stimulates expression of the HSAF biosynthetic gene (*hsaf pks*/*nrps*) and the arginase genes (*arg1* and *arg2*). Thus, spermidine affects the production of HSAF and analogs through controlling both the biosynthetic genes and the substrate-production genes.

To gain a better understanding on these two arginine-related biosynthetic pathways, we carried out in-frame deletion of *arg1* and *arg2* genes in OH11. The production of HSAF and analogs only slightly decreased in Δ*arg1* mutant or Δ*arg2* mutant (Supplementary Figure S3). However, in the double-deletion mutant (Δ*arg1*Δ*arg2*), the production of HSAF and analogs significantly decreased (Supplementary Figure S3). The results suggested that the two arginases are important for HSAF production and likely able to compensate each other in the single mutants. Notably, even the double mutant (Δ*arg1*Δ*arg2*) did not completely eliminate the production of HSAF and analogs, implied that the OH11 cells could obtain ornithine from other sources to serve as substrate for HSAF and analogs, such as from the culture media or other unknown metabolic pathways. In addition, the mutations increased slightly the cellular level of spermidine (Supplementary Figure S4). The results suggested that OH11 cells were able to maintain a proper spermidine level when the conversion from arginine to ornithine is blocked.

Many studies have shown that maintaining a proper cellular concentration of polyamines is critical to cell growth, aging, memory performance, neurodegenerative diseases, metabolic disorders and cancer (Miller-Fleming et al., 2015; Michael, 2016b; Madeo et al., 2018). Here, our data showed that cellular spermidine homeostasis is also critical to the production of a group of antifungal natural products in *L. enzymogenes*. The production of HSAF and analogs in *L. enzymogenes* is subjected to the control of a complex regulatory network with multiple signals and regulators involved. In this study, we found that the production of HSAF and analogs are markedly affected, when spermidine homeostasis is disturbed in OH11. *L. enzymogenes* OH11 uses the spermidine homeostasis to control a key substrate of HSAF and analogs. The mechanism is distinct from the previously reported protein regulator-mediated mechanisms (Qian et al., 2013; Wang et al., 2014). When the wild-type cells have too much spermidine, *arg1* and *arg2* expression would decrease; when the spermidine biosynthetic genes are mutated, exogenous spermidine would restore or even stimulate the expression of both the HSAF biosynthetic gene and the substrate ornithine-production genes.

## Conclusion

In this study, we investigated the effects of spermidine on HSAF and analogs production in *L. enzymogenes*. Our data showed that spermidine is the predominant polyamine in the cells of *L. enzymogenes* and the intracellular homeostasis of spermidine is essential for production of HSAF and analogs. We also revealed that spermidine regulated HSAF biosynthesis pathway via affecting the transcription level of HSAF key biosynthetic gene and the genes responsible for HSAF substrate conversion. In summary, our results revealed a previously unrecognized function of spermidine in regulating antifungals biosynthesis. The findings will expand our knowledge on the biological functions of spermidine.

## Author Contributions

LD, FL, and YC conceived and designed the experiments. YC and LY carried out the experiments. LD, YC, LY, and FL analyzed the data. LD, YC, and FL wrote the manuscript.

## Conflict of Interest Statement

The authors declare that the research was conducted in the absence of any commercial or financial relationships that could be construed as a potential conflict of interest.

## Supplementary Material

FIGURE S1 HPLC analysis of spermidine in wild type OH11 treated with or without exogenously supplemented spermidine (spd, 25-100 μM). Quantitative analysis of intracellular spermidine concentration is shown in the right panel. The data were derived from 3 independent experiments with triplicate samples.

FIGURE S2 HPLC analysis of spermidine concentration in wild type OH11, ΔADC, and ΔSDC treated with or without 50 μM spermidine (spd). Quantitative analysis of intracellular spermidine concentration is shown in the right panel. The data were derived from 3 independent experiments with triplicate samples. Stars indicated *P* < 0.01 compared with wild type OH11.

FIGURE S3 HPLC analysis of HASF and analogs from wild type OH11, deletion mutant of *arg1* (Δ*arg1*), deletion mutant of *arg2* (Δ*arg2*), and the double deletion mutant of *arg1* and *arg2* (Δ*arg1*Δ*arg2*). The quantitative analysis of HSAF and analogs production is shown in the right panel (***P* < 0.01). The data were derived from 3 independent experiments with triplicate samples.

FIGURE S4 HPLC analysis of spermidine concentration from wild type OH11, deletion mutant of *arg1* (Δ*arg1*), deletion mutant of *arg2* (Δ*arg2*), and the double deletion mutant of *arg1* and *arg2* (Δ*arg1*Δ*arg2*). The quantitative analysis of spermidine concentration was shown in the right panel (**P* < 0.05). The data were derived from 3 independent experiments with triplicate samples.
