# Supplementary figures and images for: Spermidine-Regulated Biosynthesis of Heat-Stable Antifungal Factor (HSAF) in Lysobacter enzymogenes OH11
Source: Front Microbiol. 2018 Dec 4;9:2984. doi: 10.3389/fmicb.2018.02984 (PMC6288370; doi:10.3389/fmicb.2018.02984)

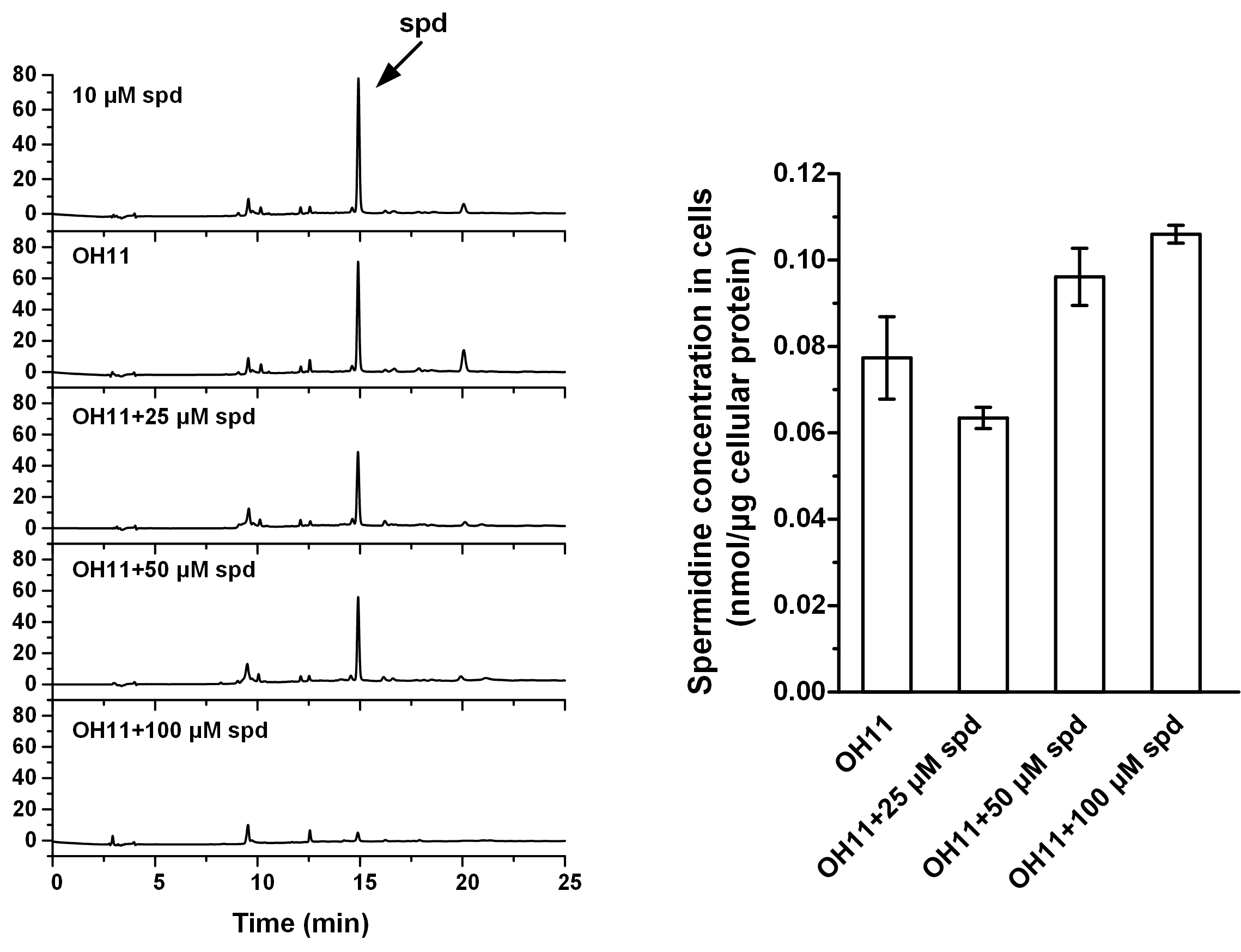

Supplement: FIGURE S1 — HPLC analysis of spermidine in wild type OH11 treated with or without exogenously supplemented spermidine (spd, 25-100 μM). Quantitative analysis of intracellular spermidine concentration is shown in the right panel. The data were derived from 3 independent experiments with triplicate samples. [file Image_1.TIF]

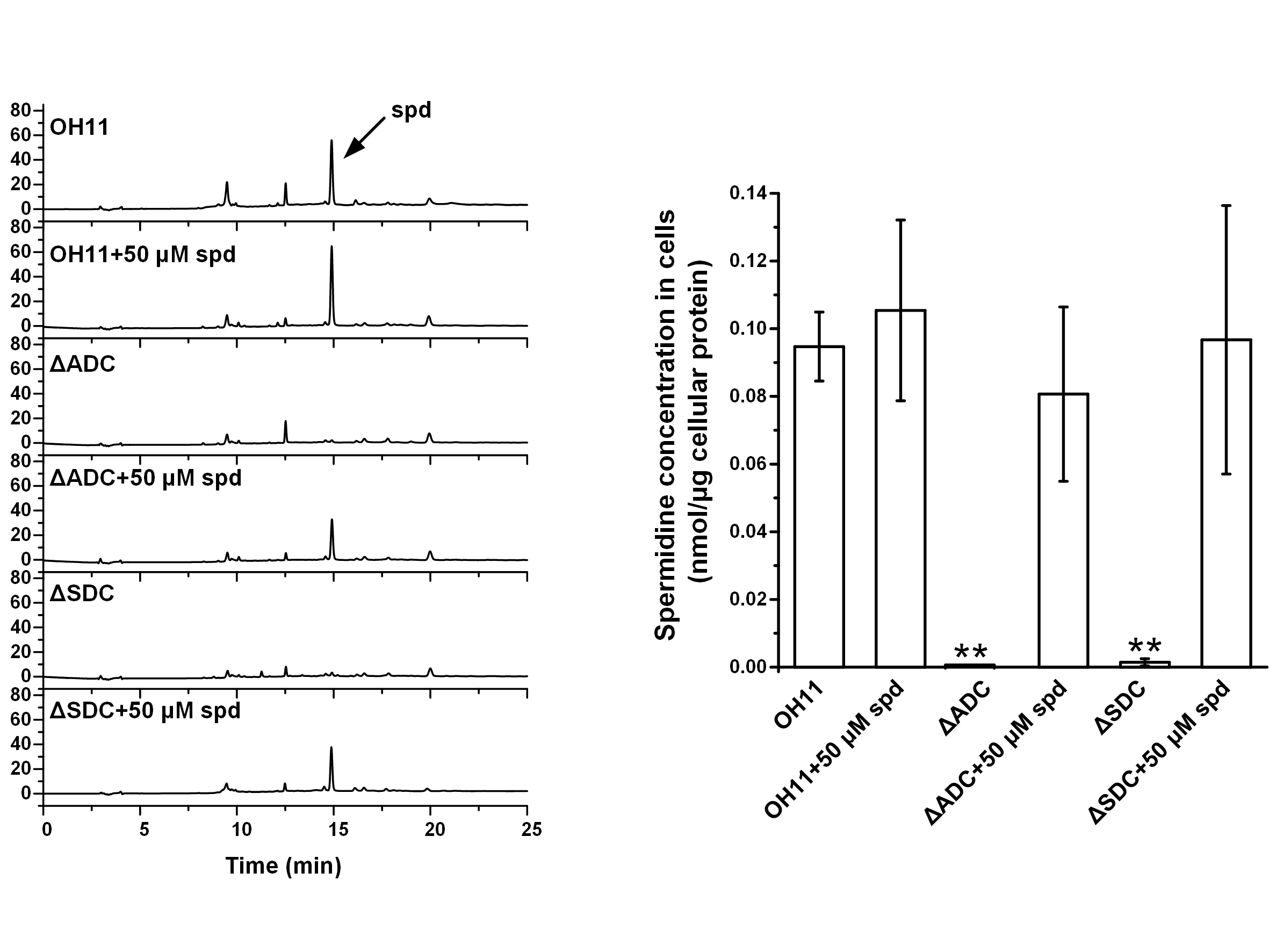

Supplement: FIGURE S2 — HPLC analysis of spermidine concentration in wild type OH11, ΔADC, and ΔSDC treated with or without 50 μM spermidine (spd). Quantitative analysis of intracellular spermidine concentration is shown in the right panel. The data were derived from 3 independent experiments with triplicate samples. Stars indicated P < 0.01 compared with wild type OH11. [file Image_2.TIF]

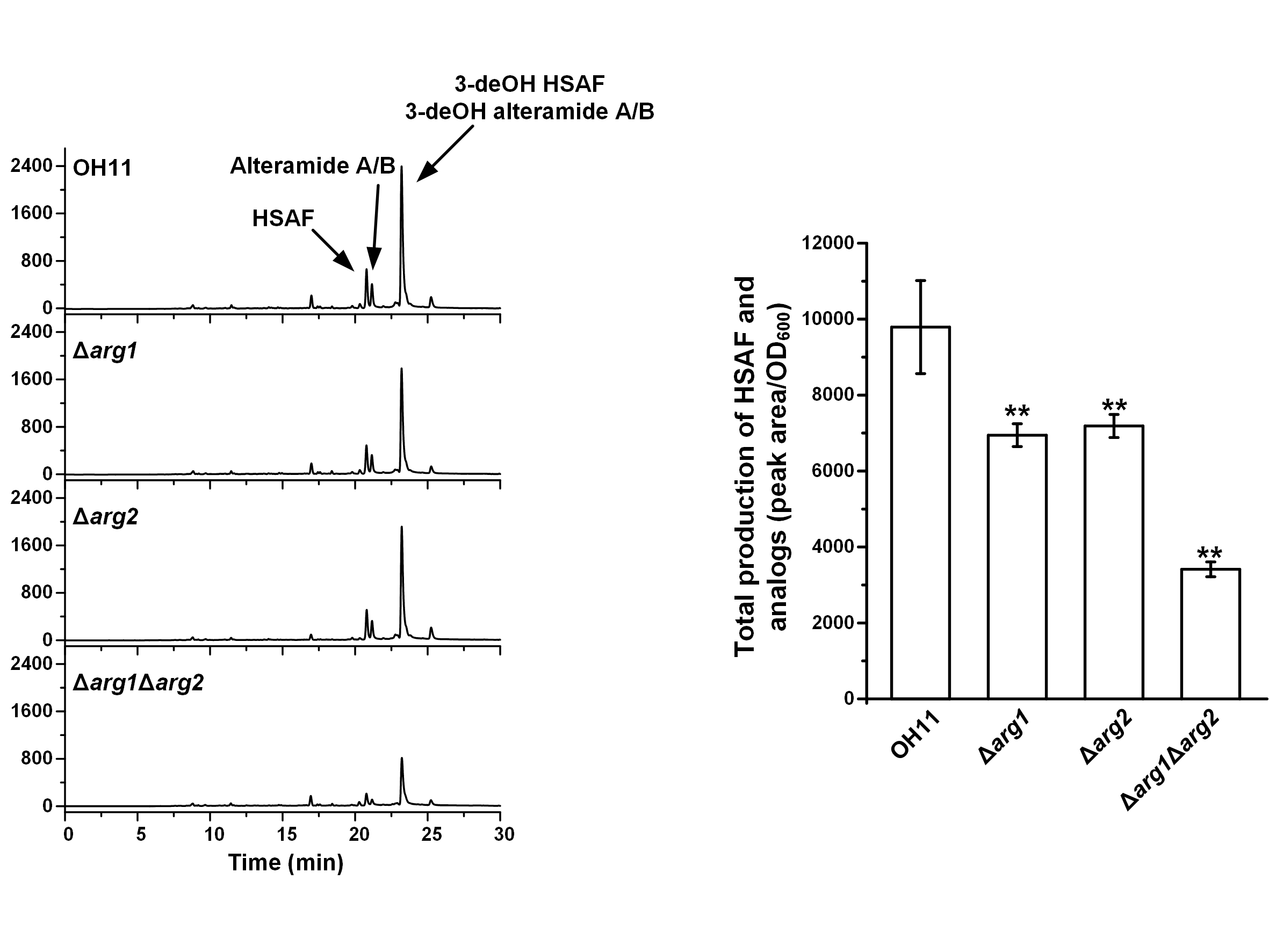

Supplement: FIGURE S3 — HPLC analysis of HASF and analogs from wild type OH11, deletion mutant of arg1 (Δarg1), deletion mutant of arg2 (Δarg2), and the double deletion mutant of arg1 and arg2 (Δarg1Δarg2). The quantitative analysis of HSAF and analogs production is shown in the right panel (**P < 0.01). The data were derived from 3 independent experiments with triplicate samples. [file Image_3.TIF]

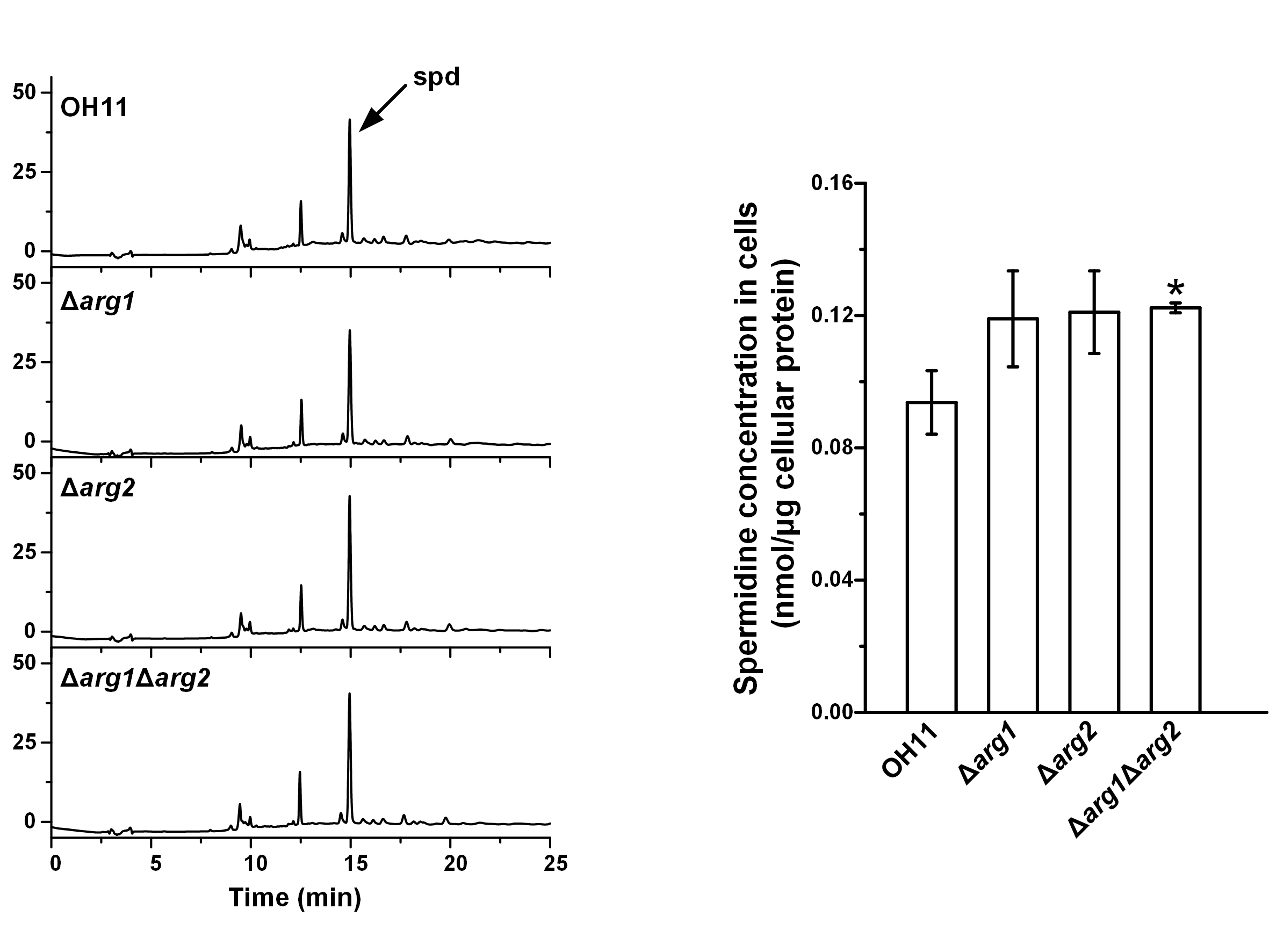

Supplement: FIGURE S4 — HPLC analysis of spermidine concentration from wild type OH11, deletion mutant of arg1 (Δarg1), deletion mutant of arg2 (Δarg2), and the double deletion mutant of arg1 and arg2 (Δarg1Δarg2). The quantitative analysis of spermidine concentration was shown in the right panel (*P < 0.05). The data were derived from 3 independent experiments with triplicate samples. [file Image_4.TIF]
